# The Effects of Peat Swamp Forest Patches and Riparian Areas within Large Scale Oil Palm Plantations on Bird Species Richness

**DOI:** 10.21315/tlsr2023.34.2.7

**Published:** 2023-07-21

**Authors:** Bettycopa Amit, Wauter Ralph Klok, Peter J. Van Der Meer, Nik Sasha Khatrina Khairuddin, Ivan Chiron Yaman, Kho Lip Khoon

**Affiliations:** 1 Malaysian Palm Oil Board, No. 6, Persiaran Institusi, Bandar Baru Bangi, 43000 Kajang, Selangor, Malaysia; 2 Van Hall Larenstein University of Applied Sciences, Larensteinselaan 26-A, 6882 CT Velp, Gelderland, The Netherlands; 3 Sarawak Oil Palms Berhad, 124–126, Jalan Bendahara, 98000 Miri, Sarawak, Malaysia; 4 Economic Planning Unit Sarawak, Chief Minister’s Department, 93502 Kuching, Sarawak, Malaysia

**Keywords:** Bird, Set-Asides Areas, Peat Swamp Forest, Riparian, Oil Palm Plantation, Biodiversity Conservation, Burung, Kawasan yang Diketepikan, Hutan Paya Gambut, Riparian, Ladang Kelapa Sawit, Pemuliharaan Biodiversiti

## Abstract

It is well established that oil palm is one of the most efficient and productive oil crops. However, oil palm agriculture is also one of the threats to tropical biodiversity. This study aims to investigate how set-aside areas in an oil palm plantation affect bird biodiversity. The research area includes two set-asides areas: peat swamp forest and riparian reserves and two oil palm sites adjacent to reserved forest sites. A total of 3,074 birds comprising 100 species from 34 families were observed in an oil palm plantation landscape on peatland located in the northern part of Borneo, Sarawak, Malaysia. Results showed that efforts by set-asides forest areas in large scale of oil palm dominated landscapes supported distinct bird species richness. High percentage of the canopies and shrub covers had a positive effect on bird species richness at area between oil palm and peat swamp forest. Herbaceous cover with height less than 1 m influenced the abundance of birds in the plantation closed to the peat swamp forest. The set-aside areas in oil palm plantations are essential in supporting bird’s refuges and should be part of oil palm landscape management to improve biodiversity conservation. Thus, provided the forest set-aside areas are large enough and risks to biodiversity and habitat are successfully managed, oil palm can play an important role in biodiversity conservation.

## Abstrak

Kelapa sawit ialah salah satu tanaman minyak yang paling cekap dan produktif. Bagaimanapun, pertanian kelapa sawit juga merupakan salah satu ancaman kepada biodiversiti tropika. Kajian ini bertujuan untuk menyiasat bagaimana kawasan terpencil di ladang kelapa sawit mempengaruhi biodiversiti burung. Kawasan penyelidikan merangkumi dua kawasan diketepikan: hutan paya gambut dan rizab riparian dan dua tapak kelapa sawit bersebelahan dengan tapak hutan simpanan. Sebanyak 3,074 ekor burung yang terdiri daripada 100 spesies daripada 34 keluarga telah diperhatikan dalam landskap ladang kelapa sawit di tanah gambut yang terletak di bahagian utara Borneo, Sarawak, Malaysia. Keputusan menunjukkan bahawa usaha oleh kawasan hutan yang diketepikan dalam landskap berskala besar yang didominasi kelapa sawit menyokong kekayaan spesies burung yang berbeza. Peratusan tinggi kanopi dan penutup pokok renek memberi kesan positif terhadap kekayaan spesies burung di kawasan kelapa sawit dan hutan paya gambut. Litupan herba dengan ketinggian kurang daripada 1 m mempengaruhi kelimpahan burung di ladang yang ditutup dengan hutan paya gambut. Kawasan yang diketepikan ladang kelapa sawit adalah penting dalam menyokong perlindungan burung dan harus menjadi sebahagian daripada pengurusan landskap kelapa sawit untuk meningkatkan pemuliharaan biodiversiti. Oleh itu, dengan syarat kawasan hutan yang diketepikan cukup luas dan risiko kepada biodiversiti dan habitat berjaya diuruskan, kelapa sawit boleh memainkan peranan penting dalam pemuliharaan biodiversiti.

**Highlights**

- Efforts by set-asides forest areas in large scale of oil palm dominated landscapes supported distinct bird species richness.
- High percentage of the canopies and shrub covers had a positive effect on bird species richness at area between oil palm and peat swamp forest while herbaceous cover with height less than 1 m influenced the abundance of birds in the plantation closed to the peat swamp forest.
- The set-aside areas in oil palm plantations are essential in supporting bird’s refugees and should be part of oil palm landscape management to improve biodiversity conservation.

## INTRODUCTION

Oil palm (*Elaeis guineensis)* is one of the most efficient and productive oil crops globally, with a production span of about 25 years (Parveez *et al*. 2020). It is a key contributor to the world edible oils and fats (World Economic Forum 2018) and plays an important role as feedstock and biofuels (Ngando-Ebongue *et al*. 2012). The impact of oil palm plantations on the environment is also significant, and the industry has faced many challenges relating to biodiversity loss and climate change (Meijaard *et al*. 2018). The Malaysian oil palm industry has countered the challenges by increasing scientific research on biodiversity conservation in oil palm production areas through set aside areas (Mohd-Azlan *et al*. 2019a; 2019b). Malaysia is also promoting sustainable practices by obliging producers to comply with the Malaysian Sustainable Palm Oil Certification Scheme (MSPO 2021).

Over the last decade, many studies have shown that oil palm agricultural expansion and intensification affected biodiversity and associated ecosystem services (Emmerson *et al*. 2016; Dislich *et al*. 2017; Guillaume *et al*. 2018). Biodiversity studies in this ecosystem often compare oil palm plantations with forests and other agriculture crops (Azhar *et al*. 2011; Yue *et al*. 2015; Hawa *et al*. 2016; Rajihan *et al*. 2017; Mitchell *et al*. 2018; Amit *et al*. 2021). In addition, other studies have focussed on different oil palm production systems and indicated that biodiversity levels are higher in smallholdings than large-scale plantations (Azhar *et al*. 2014; Syafiq *et al*. 2016; Razak *et al*. 2020). Oil palm agroecosystems biodiversity levels are affected by habitat quality which is related to a range of factors such as structural complexity, heterogeneity of vegetation cover, availability of food resources, alteration of microclimate and the human activities that lead to changes to the soil physical and chemical properties (Mariau 2001; Koh *et al*. 2009; Turner & Foster 2006; Foster *et al*. 2011; Jambari *et al*. 2012; Azhar *et al*. 2013; Drescher *et al*. 2016; Meijide *et al*. 2018). These findings showed that the negative impact of oil palm development on biodiversity could partly be mitigated by integrating the oil palm landscape with nature. Some of the recommendations to improve the level of biodiversity and its ecosystem functions includes protecting the remaining natural habitat (Phalan *et al*. 2011), increasing the structural complexity of the crop systems (Tscharntke *et al*. 2005), increasing the ground vegetation diversity of the crop landscapes (Azhar *et al*. 2013), adopting polyculture crop management strategies (Syafiq *et al*. 2016; Ghazali *et al*. 2016) and establishing set-asides of forest patches or riparian area in oil palm landscape (Mitchell *et al*. 2018; Scriven *et al*. 2019).

Recent efforts on establishing set-asides areas (e.g., wildlife corridor, forest patches, riparian area) within oil palm plantations are part of the biodiversity conservation initiatives to save wildlife (Lucey *et al*. 2014). Birds are part of the biodiversity commonly studied and found in oil palm plantations (Yudea & Santosa 2019). Birds are sensitive to any habitat disturbance and are widely used for environmental changes indicators in biodiversity conservation evaluation studies (Zakaria *et al*. 2005; Jambari *et al*. 2012; Alexandrino *et al*. 2016). Several studies have been conducted on bird diversity and its population crossing two different habitats plantation to forests, as reported by Mohd-Azlan *et al*. (2019b). However, to our knowledge, there are not much studies that examined how the vegetation structure between set-aside forest reserve and riparian within oil palm landscape affect bird diversity (Mitchell *et al*. 2018; Atiqah *et al*. 2019). Little is known about the efficiency of set-aside areas such as peat swamp forest and riparian reserve within oil palm landscapes to conserve tropical biodiversity.

This study investigates how bird species richness and abundance differed between an oil palm plantation and two set aside areas (peat swamp forest and riparian areas) in the same landscape. The study also investigates how bird species richness and abundance are associated with vegetation characteristics across a gradient of forest-edge-plantations. This paper attempts to provide detailed investigations on the importance of maintaining or creating forest areas in oil palm landscapes toward providing safe passage and refuge for birds and other wildlife while improving agriculture production.

## MATERIALS AND METHODS

### Study site

**Figure 1:**
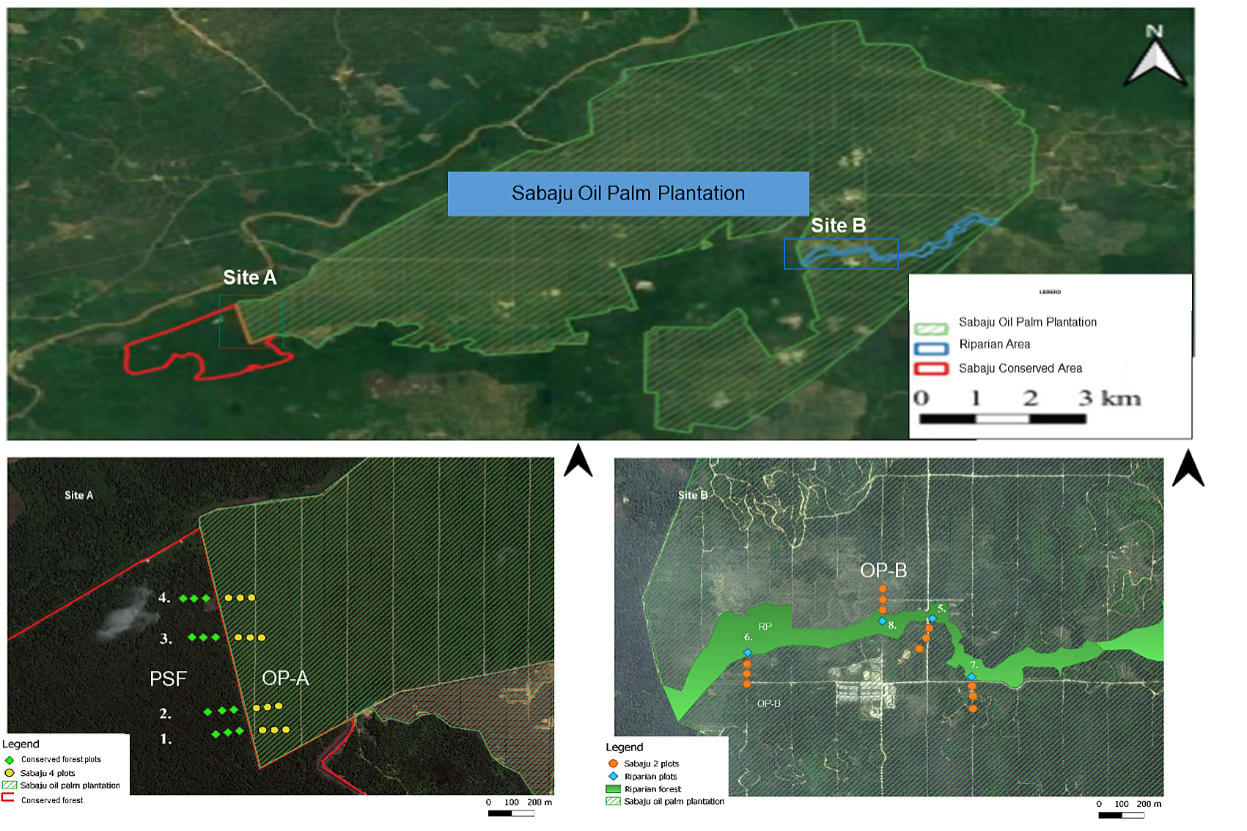
Map of the two field locations in Sabaju Oil Palm Plantation, Bintulu, Sarawak. Site A is a conserved peat swamp forest area (PSF) and Sabaju 4 Oil Palm Plantation (OP-A), while Site B is a riparian forest (RP) and Sabaju 2 Oil Palm Plantation (OP-B). The coloured dots indicate the sample plot locations for bird and habitat measurements in forest and OP plantation areas.

The study is located in Sabaju oil palm plantation (SOPP) situated in Bintulu, Sarawak, northern Borneo (N 03° 09.535” E 113° 24.640”), which belongs to Sarawak Oil Palms Berhad (SOPB). The SOPP landscapes consist of five estates: Sabaju 1, Sabaju 2, Sabaju 3, Sabaju 4 and Sabaju 5 which covers 8,116.36 ha (Fig. 1). Within this estate, two areas were set aside for conservation purposes: a peat swamp forest (PSF) and a riparian area (RP). This oil palm planting started in 2008, with the most recent planting done in 2016 (4 years after planting during sampling was carried out). SOPP is mainly located at peatland, but with some small areas on mineral soil located at Sabaju 1 and Sabaju 2.

Plantation management is according to peatland standard operating procedure (exclude mineral soil at Sabaju 1), whereby artificial drainage networks have been established to control water levels within plantations. Plantations keep their lower ground area covered with natural vegetation and minimise the use of chemicals to control the weeds. Palms located close to the riparian area were marked with red colour, indicating that this zone is free from fertiliser and chemical application. Harvesting activities of palm oil takes place twice a month inside the plantations.

**Figure 2:**
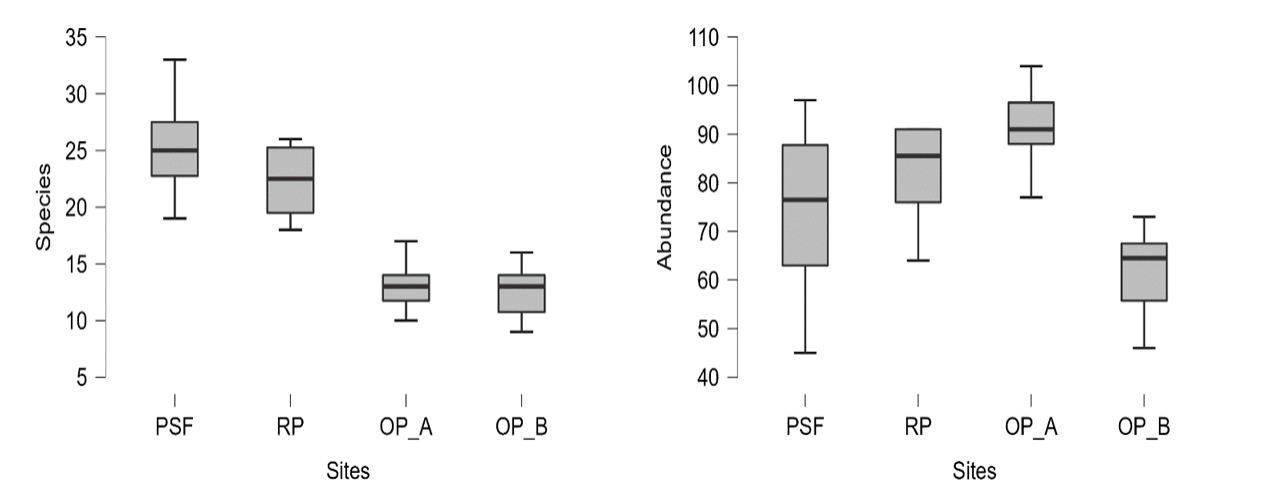
Box plot of bird species and abundance recorded in different landscapes (oil palms, riparian forest and peat swamp forest) within Sabaju oil palm plantation.

Sampling was conducted in 2020 at the two set-aside areas and adjacent OP plantations (Fig. 2):

1. Site A is a patch 343 ha of conserved PSF located at the south-west part of SOPP. This forest previously was logged PSF, but now this forest was preserved and protected from any other development for scientific research and conservation efforts by the plantation owner. The adjacent OP is Sabaju 4, which covers an area of 1,831 ha, planted between 2010 to 2011 and the study site for OP-A located at palm aged 9 years old after planting during sampling was carried out. Ground vegetation of OP-A consists mostly of ferns.
2. Site B is a small (< 50 ha) patch of RP situated at the eastern part of SOPP. This forest grows on the banks of the Sujan River that crosses the SOPP. These forests are conserved as a buffer zone between SOPP and the river. The buffer zone is implemented to avoid leakage of fertiliser and chemicals into the river’s ecosystem, thus preserving the water quality. The RP is located in the Sabaju 2. Sabaju 2 (OP-B) covered an area of 2,282 ha, and the palm ranged from 7–12 years after planting. The herbaceous cover at sampling site for OP-B is dominated mainly by grasses and has wetter conditions with more irrigation canals running through the plantations.

### Habitat and Bird Sampling

#### Habitat measurements

Vegetation parameters measured at each plot according to Rodwell (2006) showed at Table 1.

#### Bird observations

The distance point count technique was used at each plot to observe the bird species (Buckland *et al*. 2001; Zakaria *et al*. 2009). Four transect lines of 300 m were set up at site A, covering 150 m of peat swamp forest (50 m, 100 m and 150 m) and 150 m of OP plantation (50 m, 100 m and 150 m) from the forest edge. Six observation plots were established per transect at intervals of 50 m. The wetter conditions at Site B inside the riparian forest prevented setting up plots approximately 10 m inside the riparian forests. Consequently, the four transect lines were only 160 m in length covering 10 m of riparian forest and 150 m of OP plantation (50 m, 100 m and 150 m) from the forest edge. Counts were conducted from 0630 am–1100 am, and each observation plot was sampled for 20 min. To avoid time-of-day biases, the plots were visited four times in alternate order for each transects. Data on the transects are not independent and have been analysed for trends linked with distance from forest edge. However, the distance between transects was more than 100 m and we assume that individual birds recorded at 1 transect were not recorded at the other transect. Only species heard and sighted within the 20 m radius from the points’ counts plot were recorded as present. Bird calls were recorded using Rode VideoMic GO attached to a Nikon D3300 camera. The information on present bird species was obtained from the *Birds of Borneo* handbook by Myers (2009). Bird vocalisations were used to locate birds and to aid identification.

### Statistical Analysis

One-way analysis of variance (ANOVA) and linear regression were computed using JASP Version 0.16.2 (JASP Team 2022). One-way ANOVA was used to compare bird species richness and abundance among sites. In testing for significant differences between sites RP (4 plots) was excluded from the analysis due to unequal plots in comparison to the other three sites (12 plots). However, we maintain RP in the boxplot figure as it shows mean of sites. In addition, this analysis was also used to compare the percentage of canopy cover, shrub cover and herb cover between sites. Tukey post hoc test was used to explore multiple comparison of mean differences of measures parameters (species richness, species abundance, percentage of canopy cover, percentage of shrub cover and percentage of herb cover) between sites. The linear regression was used to correlate between bird communities and vegetation structure in Sabaju oil palm plantation.

## RESULTS

### Overall Bird Species Richness and Abundance in Oil Palm Landscape

A total of 3,074 birds belonging to 100 species and 32 families were observed in SOPP (Table 2). Seventy-seven species were recorded in the PSF, 45 species in the RP, 31 species in the OP-A, and 30 species in the OP-B, including one peat swamp forest species, Hook-billed Bulbul (*Setornis criniger*) and one endemic to Borneo (*Dusky Munia (Lonchura fuscans*). Overall, twenty conservation priority species (Table 1) were recorded in the SOPP whereby the PSF recorded 15 species, RP with five species, OP-A with four species and OP-B with three species.

**Figure 3:**
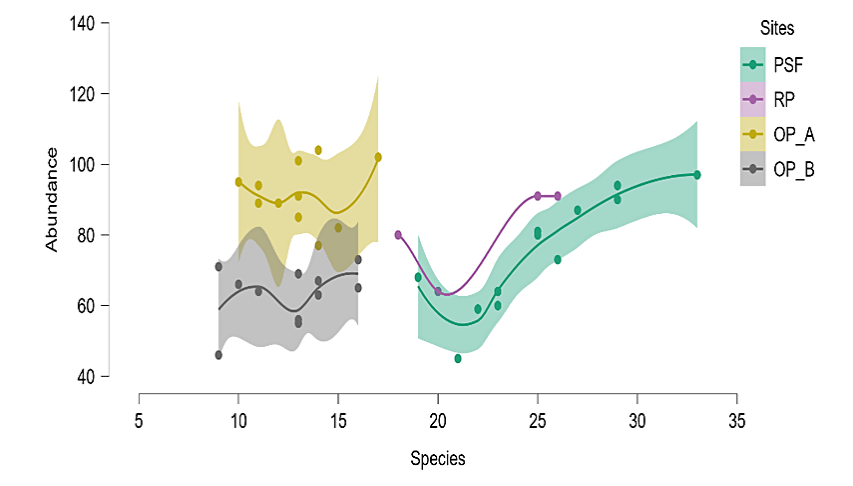
Scatter plot of bird species and abundance recorded in different landscapes (oil palm areas, riparian forest and peat swamp forest) within Sabaju oil palm plantation). The highlighted areas in the figure show the scatter of the data for the PSF and the two OP sites. No scatter for RP is shown because of limited number (4) of plots.

One-way ANOVA showed a significant effect of the different habitat types in oil palm landscape on bird (F_(3, 36)_ = 50.24, *p* < 0.001, ω^2^ = 0.787). Post hoc testing using Tukey’s revealed that set-asides areas PSF (mean = 25 species) significantly (*p* < 0.001) high species richness than in oil palm areas [OP_A (mean = 13) and OP_B (mean = 12)]. RP site has been excluded from testing using one-way ANOVA to compare species richness and abundance due to unequal sampling points with PSF, OP_A and OP_B. There was no significant different bird species richness between OP_A and OP_B (*P* = 0.998). Hence, set-aside areas do support high number of bird species richness in oil palm plantation. Also, the abundance of birds was showed a significant effect of the different habitat landscape in the oil palm plantations (F_(3, 36)_ = 13.26, *p* < 0.001), ω^2^ = 0.479). Post hoc testing showed that OP_A showed significantly more bird abundance than PSF and OP_B (*p* < 0.001). Scatter plots showed that set-asides areas recorded high number of species with less bird abundance while for oil palm areas recorded high bird abundance with less number of species (Fig. 3).

Yellow-vented Bulbul (*Pycnonotus goiavier*) was the most dominant species with 817 individuals followed by Plain Sunbird, *Anthreptes simplex* with 328 individuals, Ashy Tailorbird *Orthotomus ruficeps* with 198 individuals and Pied Fantails *Rhipidura javanica* with 172 individuals and all of these species recorded in all sites (PSF, RP, OP-A and OP-B). The group of babblers represented the largest number of species with 17 species whereby recently has been divided into two families (Timaliidae and Pellorneidae) by Cai *et al*. (2019) through DNA sequencing method. This was followed by Family Nectariidae (spiderhunters and sunbirds) with 13 species. More than 90% of species from these three families were recorded at the PSF. For RP, Family Nectaridae was the most dominant family with seven species, followed by Timaliidae with five species. OP-A that closed to the peat swamp forest showed a high number of species from the family Nectariidae (sunbirds) with eight species followed by Dicaeidae (flowerpeckers) with four species. OP-B that closed to the riparian area recorded mostly from the family Ardeidae (egrets, bitterns) with five species followed by Cisticolidae (prinias and tailorbirds) with four species. Good presentation of family Ardeidae related to the presence of waterbody or river along the riparian area.

### Bird and Vegetation Structure at Forest and Plantation in Relation to Distance to The Forest Edge

In this study, six species have been recorded in both sites (Site A and Site B) with different distance from forest edge (Table 2); Plain Sunbird, Yellow-vented Bulbul, Malaysian Pied Fantail, Orange-bellied Flowerpecker, Ashy Tailorbird and Yellow-bellied Prinia. In Site A, five out of nine species have been recorded in all distance from forest edge showed relative abundance more than 5% (Yellow-vented Bulbul: 33%; Plain Sunbird: 8%; Ashy Tailorbird: 7%; Brown-throated Sunbird and Malaysian Pied Fantail: 5% each) from the total abundance of bird species recorded (1998 individuals). In Site B, seven out of 12 species have been recorded in all distance from forest edge showed relative abundance more than 5% (Plain Sunbird: 16%; Yellow-vented Bulbul: 15%; Oriental Magpie Robin: 10%; Orange-bellied Flowerpecker: 8%; Malaysian Pied Fantail and Dusky Munia: 7% each; Yellow-bellied Prinia: 6%; Ashy Tailorbird: 5%) from the total abundance of bird species recorded in Site B (1,076 individuals).

**Figure 5:**
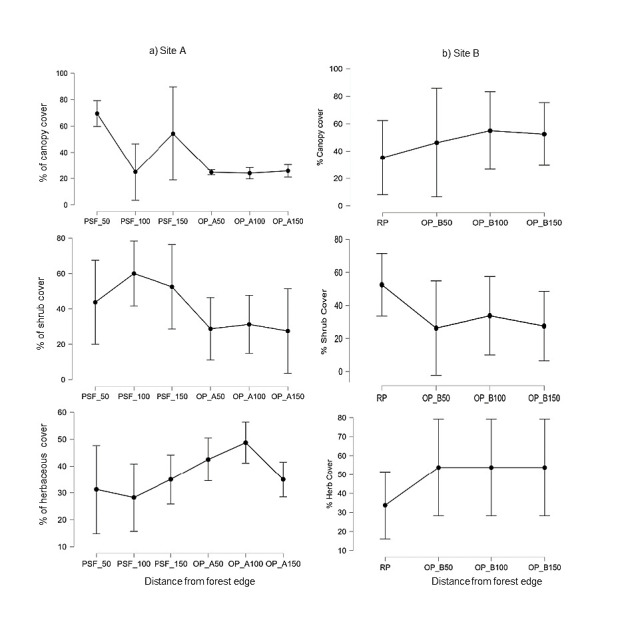
Percentage of canopy cover, shrub cover and herb cover at different distance from the edge (50 m, 100 m and 150 m) for (a) Site A (peat swamp forest reserved (PSF) and oil palm plantation (OP-A), and (b) Site B (riparian reserves (RP distance to the edge 10 meters)-edge-oil palm plantation (OP-B)).

Bird species richness (Fig. 5) differed significantly among distances of PSF and OP-A to the edge (F_(5, 18)_ = 20.00, *p* < 0.001). The highest bird species richness occurs in PSF closer to the edge (mean: 50 m = 27 species per sampling point), while the lowest occurred interior to the OP-A (mean: 100 m and 150 m = 12 species per sampling point. The different distances (50 m, 100 m and 150 m) of PSF and OP-A to the edge had similar levels of species abundance (F_(5, 18)_ = 2.86, *p* = 0.05). Across all transects, the highest value for species abundance in OP-A was 100 m to the edge (mean = 94 birds), whereas the lowest occurrence was at interior of the PSF (mean: 150 m = 84 birds). There was no significant difference in bird species richness and abundance between 50 m to 150 m of PSF or OP-A to the edge. Our results showed that bird species richness (F_(3, 12)_ = 12.42, *p* = 0.001) and abundance (F_(3, 12)_ = 4.44, *p* = 0.03) are different between distances of RP and OP-B to the edge. RP supported greater bird species richness (mean = 22 species per sampling point) and abundance (mean = 82 birds) than any distances of OP-B to the edge. The lowest species richness was recorded in OP-B at 150 m to the edge (mean = species), and the lowest species abundance was recorded in O-PB at 100 m to the edge (mean = 58 birds).[Fig f4-tlsr-34-2-131]

In terms of vegetation structure (Fig. 5), percentage of canopy (F_(5, 18)_ = 12.758, *p* = 0.000), shrub (F_(5, 18)_ = 4.299, *p* = 0.009) and herb cover (F_(5, 18)_ = 5.147, *p* = 0.004) had significantly differed among different distance of PSF and OP-A to the edge. PSF revealed high percentage of canopy and shrub cover but low percentage of herbaceous cover than in any distance of OP-A to the edge. PSF at 50 m closer to the edge recorded high percentage of canopy (mean: 50 m = 69.5%), while shrub cover was recorded high at 100 m to the edge (mean = 60%). OP-A recorded high percentage of herbaceous cover but low percentage of canopy and shrub cover recorded. There were no significant difference in terms of percentage of canopy (F_(3, 12)_ = 0.862, *p* = 0.487), shrub (F_(3, 12)_ = 2.725, *p* = 0.091) and herb (F_(3, 12)_ = 1.794, *p* = 0.202) cover among different distances of RP and OP-B TO the edge. RP recorded high percentage of shrub cover (mean = 52.5%) but low percentage of canopy (mean = 35.25%), and herbaceous cover (mean = 33.25%). In term of OP-B, high percentage of canopy (mean in the range between 50 m–150 m to the edge = 46.25%–55%) and herbaceous (mean between 50 m–150 m to the edge = 53.75%) but low percentage of shrub cover (mean in the range between 50 m–150 m to the edge = 26.25%–33.75%) were recorded.

### Effect of Peat Swamp Forest and Riparian Reserves in Oil Palm Agroecosystem to Bird Community

**Figure 6:**
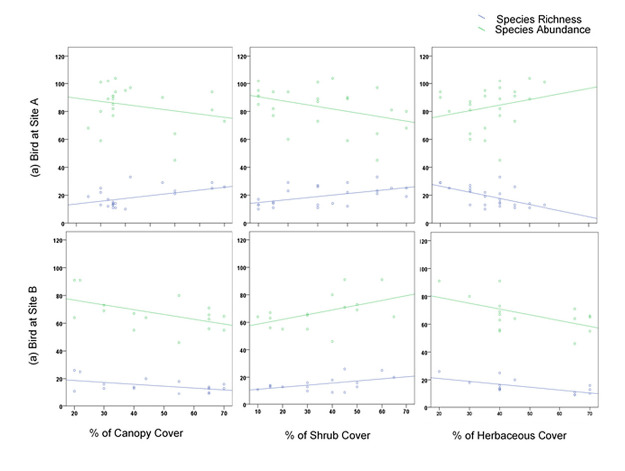
Linear regression analysis of the relationship between bird and vegetation structure including the percentage of canopy cover, percentage of shrub cover and percentage of herb cover at (a) Site A: transition from peats swamp forest (PSF) – forest edge-oil palm plantation (OP-A), and (b) Site B: transition from riparian reserve (RP) – forest edge-oil palm plantation (OP-B).

Our study indicated a strong relationship between the vegetation variables and the species richness and abundance of birds at Site A which across gradient from forest edge to interior of plantation or peat swamp forest (Fig. 6). The percentage of canopy (R^2^= 0.389, F_(2,21)_ = 6.672, *p* = 0.006), shrub (R^2^ = 0.357, F_(2,21)_ = 5.822, *p* = 0.01) and herbaceous cover (R^2^ = 0.348, F_(2,21)_ = 5.609, *p* = 0.011) had significant effect on the bird species richness and abundance. The regression results indicate that bird species richness was positively related to canopy and shrub cover but negatively related to herbaceous. The results showed that bird species richness in Site A increased with a high percentage of canopy or shrub cover and decreased in the percentage of herbaceous cover. Bird species abundance indicated positive relation with herbaceous cover and negative relation with canopy and shrub cover. Bird species abundance increased with a high percentage of herbaceous cover and decreasing percentage of canopy and shrub cover.

A linear regression analysis of bird species richness and species abundance in relation to RP and OP-B at Site B is shown in Fig. 6. Bird species richness and abundance negatively correlated with herbaceous cover (R^2^ = 0.435, F_(2,21)_ = 4.997, *p* = 0.025), whereby bird species richness higher with decreasing herbaceous cover. Percentage of canopy (R^2^ = 0.299, F_(2,13)_ = 2.777, *p* = 0.099) and shrub (R^2^ = 0.277, F_(2,21)_ = 2.486, *p* = 0.122) had no significant effect on bird species richness and abundance.

## DISCUSSION

Overall species richness showed that PSF recorded a high number of species with 77 species (mean = 25 species/point) followed by RP with 45 species (mean = 22 species/point) than plantations (OP-A: 31 (mean = 13 species/point) species and OP-B: 30 species (mean = 12 species/point)). The high number of species in PSF than RP might be due to the size of PSF which is broad in shape and connected with adjacent forest while RP is narrow-line-shaped along the river line. Narrow-linear shaped forest areas within the oil palm landscape did not support high species richness (Mohd-Azlan *et al*. 2019b). Connecting habitat with the nearby forest is crucial (Hawa *et al*. 2016; Lindenmayer & Fischer 2013) to create wildlife landscape connectivity to support high biodiversity value within oil palm landscapes. This study showed that set-asides areas recorded high number of species with less bird abundance while for oil palm areas recorded high bird abundance but low number of species and this finding like Aratrakorn *et al*. (2006) and Amit *et al*. (2021) found less to high abundance of birds across the conversion of lowland forest or logged peat swamp forest to oil palm plantation.

Fragmented and isolated forest patches are often assumed to have low conservation value because their species communities are depauperate, and important ecosystem services may be reduced or absent in small fragments (Miller-Rushing *et al*. 2019). However, based on the results of this study, set-aside areas: RP and PSF recorded high number of bird species richness within the oil palm dominated landscape. Results for RP is consistent with what was found in a previous study by Mitchell *et al*. (2018), stating that riparian reserves help protect forest bird communities in oil palm dominated landscapes. Forested sites (PSF, and also RP) supported greater bird diversity than the plantation sites: this may well be linked to the complexity of the forest structure, supporting greater flora diversity and providing better habitat and greater food resources than the plantation sites (Turner & Foster 2009; Yule 2010; Azhar *et al*. 2011; Posa 2011; Hawa *et al*. 2016). Mansor and Sah (2012) study showed that forest patches key factor to provide foraging opportunities for birds even though bird’s foraging behaviour may show differential responses in this habitat due to compete more intensely with each other for the remaining resources. However, this finding contradicts with the findings by Mohd-Azlan *et al*. (2019b) through the mist-netting method. The narrow linear shape forest area within oil palm dominated landscape could not support bird species diversity, and it is similar for plantation area as compared to forest edge which recorded higher bird species diversity.

Interestingly, these set-asides areas, PSF and RP within oil palm landscape provide refuge for threatened species, specialist species to PSF, and bird species endemic to Borneo hence indirectly some of these species such as Short-tailed Babbler, Black-throated Babbler, Black Hornbill and Dusky Munia also recorded in oil palm plantation area. This finding was consistent with previous studies that also recorded threatened species in oil palm plantations (Sheldon *et al*. 2010; Azhar *et al*. 2011; Mohd-Azlan *et al*. 2019b; Amit *et al*. 2021). Plantations that recorded threatened, migratory, forest and wetland species have some conservation value within oil palm dominated landscape (Azhar *et al*. 2011). These results indicated how forest patches give conservation values in oil palm dominated landscapes. It plays an important role as a bird diversity hotspot and refuge for threatened species, thus sustaining biodiversity in Borneo. Mansor *et al*. (2019) also mentioned that continuous forest has critically important characteristics that need to be conserved similar goes to forest patches are also important as ecological movement corridors and foraging ground for birds. PSF recorded a high number of threatened species in the world as compared to RP. The primary contributor was the location of PSF connecting with adjacent forest. Scriven *et al*. (2019) noted that habitat connectivity is important to support threatened and forest-dependent species for improving tropical biodiversity conservation.

Due to its richness in PSF, Family Nectariniidae also recorded higher in plantations close to it but not for Family Timaliidae. Zakaria *et al*. (2005) mentioned that the Family Nectariidae are known as habitat colonisers and common in disturbed areas, while Family Timaliidae is mostly forest birds that are more sensitive to habitat disturbance (Moradi & Mohamed 2010). PSF continued to support and provide habitat for birds that are sensitive to disturbance such as forest birds like the Family Timaliidae, even though this area is located within the oil palm plantation. However, in comparison with plantations close to RP, wetland birds from the Family Ardeidae were the richest and attracted to wetland close to the river. These results are in line with the previous study by Azhar *et al*. (2013) who showed that wetland birds move more in plantations due to the presence of wetland habitats such as ponds and drainage as an aquatic habitat that provides food resources to these birds. Hence, wetland birds are also attracted to riparian reserves within the oil palm dominated landscapes.

### Study of Birds for Gradient Distance from Forest to Oil Palm Plantation

In this study, at Site A bird species richness and species abundance were significantly different among all distances of 50 m, 100 m and 150 m from the edge to the interior peat swamp forest or oil palm plantation whereby high species richness at forest closed to the edge (PSF 50 m) and species abundance recorded higher interior oil palm plantation (OP-A at distance 100 m and 150 m). Even though the present study uses the point count method, the findings seem to be consistent with other research using the mist-netting method, which found significant differences in bird diversity across the gradient from interior forest and plantation to the edge (Mohd-Azlan *et al*. 2019b). Mohd-Azlan *et al*. (2019b) study was conducted at the edge and different distances of 100 m, 200 m and 300 m from the edge to the interior plantation or forest. Results from that study showed that the edge and interior forest recorded higher species richness than the interior plantation. This study (Mohd-Azlan *et al*. 2019b) showed that edge and interior forest recorded higher species richness than interior plantation due to the edge effect. Edge effect is defined as changes that occur at the abrupt transition between adjacent habitats resulting from the juxtaposition of contrasting ecosystems on either side of the discontinuity (Sammalisto 1957). The edges contain increased biodiversity since they attract species which are able to exploit both sides of the discontinuity in addition to those species’ characteristics of either side (Clapham 1973). The presence of bird species that can exploit both or either side are influenced by food specialisation and habitat association which include humidity, light intensity and temperature (Maina 2002) and canopy density (Stone *et al*. 2018). However, this study observation was not conducted at the edge site, but the results showed that forest at 50 m from the edge recorded higher species richness than interior forest (100 m and 150 m). This finding indicates that edge effect might still apply at 50 m forest from the edge, as compared to 100 m and 150 m of forest to the edge, with lower species richness.

### The Effect of Vegetation Structure to Bird Species Richness and Abundance at Different Distances to the Forest Edge

This high percentage of canopy and shrub cover supported greater bird species richness in PSF than in interior plantation reflects the findings of Azhar *et al*. (2013). This study also suggested that the higher density of shrub covers in RP supported a higher value of species richness and abundance of birds. This finding is similar to previous research (Mitchell *et al*. 2018), suggesting that RP in oil palm plantations supported distinct bird communities. Due to the structure of the canopy layer, PSF and RP attracted forest bird species such as trogons, iora, barbet and broadbills and arboreal birds such as pigeons and bee-eater. These findings are in line with the results of Peh *et al*. (2006), Hawa *et al*. (2016) and Beskardes *et al*. (2018). In addition, some species prefer higher canopy closer to the forest edge for predation such as eagles, dollar birds, hornbill and falconet. These species prefer taller trees as a lookout for prey (Andersson *et al*. 2009). During observation, black hornbill was spotted feeding on beetles on the top of tall trees in RP. Furthermore, the canopy of the PSF provides sufficient sunlight and space for the development of shrub layers such as lianas, epiphytes and hemiepiphytes which may attract forest birds such as babblers, prinias, bulbuls and sunbirds that utilise the different vegetation strata (Gaither 1994; Peh *et al*. 2006; Azhar *et al*. 2013; Hawa *et al*. 2016; Mansor & Ramli 2017; Mansor *et al*. 2019). Study by Mansor *et al*. (2019) reported the important of aerial curled dead leaves within the aboveground vertical vegetation layers in the forest as a foraging area for a group of babblers. Negative relation between bird species richness and abundance with herbaceous cover at Site B might be due to herbaceous habitat structure at OP-B closed to RP was dominated by grass and has wetter condition which attract more wetland birds such as egrets, bitterns and waterhen.

However, this study showed that the increasing percentage of herbaceous cover and decreasing percentage of canopy and shrub cover has a higher abundance of birds in the interior of the plantation than in PSF. Oil palm stands on peat that was nine years (OP-A) have open canopy cover which provides direct sunlight for the development of ground vegetation layers such as herbaceous mostly at oil palm circles, frond pile and harvesting paths, while shrub cover was along the field drain. A bit different from oil palm plantation close to RP, the percentage of herbaceous and canopy cover was higher than in OP-A. High canopy cover was due to the palm age (11-year-old). The increased canopy cover still provides enough sunlight for the development of herbaceous cover. The low percentage of shrubs than herbaceous in oil palm plantations might be due to the systematic weeding practices, and this finding is consistent with Azhar *et al*. (2011). The abundance of birds in plantation is due to the presence of dominant species such as Yellow-vented Bulbul, Oriental Magpie Robin, Malaysian Pied Fantail, Plain Sunbird, and Ashy Tailorbird. The results of this study are consistent with Hawa *et al*. (2016), who reported these birds’ species in oil palm plantations. Amit *et al*. (2015) also mentioned that the most dominant species in oil palm plantation was Yellow-vented Bulbul and stated that this species feed on oil palm pollinators weevil such as *Elaeidobius kamerunicus*. The abundance of these birds relies on the thickness of the vegetation cover, which provides refuge from predators which possibly related to their anti-predator strategies and provides food resources (such as arthropods and seeds) (Azhar *et al*. 2011; Tamaris *et al*. 2017; Ashton-Butt *et al*. 2018). Also, these species provide essential ecosystem services for plantations to control pests such as bagworms (Chennon & Susanto 2006; Koh 2008; Hawa *et al*. 2016). Hence, it is important to maintain vegetation cover to support the survival of these birds’ species in oil palm plantations.

## CONCLUSION

Our results demonstrate a strong effect of set-asides areas; peat swamp forest and riparian area supported bird species richness and abundance in overall oil palm dominated landscapes. High canopy and shrub cover by maintaining forest patches in oil palm landscape provides habitat for the forest, wetland, endemic, predator and threatened species of birds. Nevertheless, we found that a high percentage of herbaceous cover may result in high abundance of birds in the oil palm area closed to peat swamp forest. Requirements of protecting and conserving the concerned species are the most important strategies that should be supported through better management of the set-aside areas within oil palm dominated landscape hence producing sustainable palm oil production. Set-aside areas should be linked and connected with nearby forests to improve wildlife landscape connectivity. Future research should highlight how bird species in set-aside areas provide ecosystem services to the interior of the oil palm landscapes.

## Figures and Tables

**Figure 4 f4-tlsr-34-2-131:**
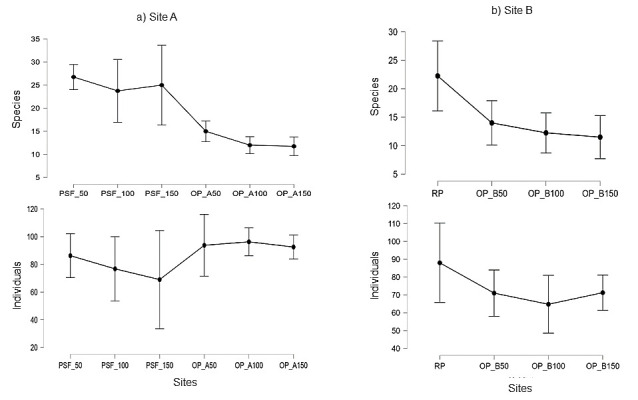
Bird species and individuals at different distance from the edge (50 m, 100 m and 150 m) for (a) Site A (peat swamp forest reserved (PSF)-edge-oil palm plantation (OP-A), and (b) Site B (riparian reserves (exclude RP the distance from the edge to RP 10 m)-edge-oil palm plantation (OP-B)).

**Table 1:** Vegetation parameters measured at each plot.

Vegetation parameters	Description	Measurement method
Canopy cover	The upper layer of the forest dominated by tree species	Percentage of canopy coverage with the use of Canopyapp, programmed by the University of Hampshire (2018). At each location, four pictures of the canopy were taken in the four cardinal directions. The average of these percentages is used as the canopy cover of the observation plot.
Shrub cover	The woody vegetation layer between 1 m in height and the tree canopy	The shrub cover was estimated by selecting four sampling plots measuring 4 m *×* 4 m in the observation plot. The average shrub growth in each sampling plot is used as the total shrub coverage for the observation plot.
Herbaceous cover	The non-woody vegetation layer less than 1 m tall	The herbaceous cover was estimated with the use of four sampling plots measuring 1 m *×* 1 m. the average coverage of the sampling plots is used as the overall coverage of herbaceous vegetation in each observation plot.

**Table 2:** List of birds recorded at different landscapes including peat swamp forest reserves (PSF), Riparian area (RP) and oil palm (OP) with their conservation status (VUL: Vulnerable; NT: Near Threatened; LC: Least Concern) according to Red List of Near Threatened species under International Union for Conservation of Nature (IUCN) in Sabaju Oil Palm Plantation, Bintulu, Sarawak.

Family	Species name	Scientific name	Sites according to distance from the edge										

			PSF 50	PSF 100	PSF 150	RP	OP_A 50	OP_A 100	OP_A 150	OP_B 50	OP_B 100	OP_B 150	IUCN status
Nectariniidae	Spectacled Spiderhunter	*Arachnothera flavigaster*	/	/	/	/		/	/				LC
	Little Spiderhunter	*Arachnothera longirostra*	/	/	/	/		/	/	/	/		LC
	Ruby-cheeked Sunbird	*Chalcoparia singalensis*	/	/	/		/	/					LC
	Purple-naped Sunbird	*Arachnothera hypogrammica*	/		/	/	/	/					LC
	Plain Sunbird	*Anthreptes simplex*	/	/	/	/	/	/	/	/	/	/	LC
	Purple-throated Sunbird	*Leptocoma sperata*	/			/	/						LC
	Brown-throated Sunbird	*Anthreptes malacensis*	/	/	/		/	/	/				LC
	Red-throated Sunbird	*Anthreptes rhodolaemus*	/		/		/		/				LC
	Olive-backed Sunbird	*Cinnyris jugularis*	/		/	/							LC
	Temminck’s Sunbird	Aethopyga temminckii		/									LC
	Crimson Sunbird	*Aethopyga siparaja*		/									LC
	Copper-throated Sunbird	*Leptocoma calcostetha*				/							LC
	Yellow-eared Spiderhunter	*Arachnothera chrysogenys*			/								LC
Timaliidae	Chestnut-backed Scimitar Babbler	*Pomatorhius montanus*	/										LC
	Bold-striped Tit-babbler	*Mixornis bornensis*	/		/	/						/	LC
	Chestnut-rumped Babbler	*Stachyris maculata*	/										NT
	Black-throated Babbler	*Stachyris nigricollis*	/	/	/		/						NT
	Chestnut-winged Babbler	*Cyanoderma erythropterum*		/	/								LC
	Grey-headed Babbler	*Stachyris poliocephala*			/								LC
	Fluffy-backed Tit-babbler	*Macronus ptilosus*	/	/	/								NT
	Grey-throated Babbler	*Stachyris nigriceps*			/								LC
	Rufous-fronted Babbler	Cyanoderma rufifrons				/							LC
Pellorneidae	Horsfield’s Babbler	*Trichastoma sepiarium*	/										LC
	White-chested Babbler	*Trichastoma rostratum*	/	/		/							NT
	Black-capped Babbler	*Pellorneum nigrocapitatum*	/	/	/	/		/					LC
	Short-tailed Babbler	*Trichastoma malaccense*	/	/	/							/	NT
	Rufous-crowned Babbler	*Malacopteron magnum*	/	/	/								NT
	Sooty-capped Babbler	*Malacopteron affine*	/	/	/								NT
	Moustached Babbler	*Malacopteron magnirostre*		/									LC
	Abbott’s Babbler	*Malacocincla abbotti*	/			/							LC
Pycnonotidae	Yellow-vented Bulbul	Pycnonotus goiavier	/	/	/	/	/	/	/	/	/	/	LC
	Black-headed Bulbul	*Pycnonotus atriceps*	/	/									LC
	Hook-billed Bulbul	*Setornis criniger*	/			/							VU
	Olive-winged Bulbul	*Pycnonotus plumosus*	/	/	/	/	/	/		/	/	/	LC
	Puff-backed Bulbul	*Pycnonotus eutilotus*		/									NT
Picidae	Rufous Woodpecker	*Celeus brachyurus*	/	/	/	/							LC
	White-bellied Woodpecker	*Dryocopus javensis*	/										LC
	Olive-backed Woodpecker	*Dinopium rafflesii*	/	/									NT
	Buffed-rumped Woodpecker	*Meiglyptes grammithorax*			/								LC
	Common Flameback	*Dinopium javanense*			/								LC
	Rufous Piculet	*Sasia abnormis*	/		/								LC
Dicaeidae	Orange-bellied Flowerpecker	*Dicaeum trigonostigma*	/	/	/	/	/	/	/	/	/	/	LC
	Yellow-breasted Flowerpecker	*Prionochilus maculatus*	/	/	/	/	/	/	/				LC
	Scarlet-breasted Flowerpecker	*Prionochilus thoracicus*	/	/	/								NT
	Yellow-rumped Flowerpecker	*Prionochilus xanthopygius*	/	/	/	/			/	/	/	/	LC
	Yellow-vented Flowerpecker	*Dicaeum chrysorrheum*			/								LC
	Yellow-bellied Flowerpecker	*Dicaeum melanozanthum*					/						LC
Sturnidae	Hill Myna	*Gracula religiosa*	/	/	/	/							LC
Musicapidae	Oriental Magpie-robin	*Copsychus saularis*	/			/	/	/	/	/	/	/	LC
	Malaysian Blue Flycatcher	*Cyornis turcosus*					/	/	/				NT
	Grey-chested Jungle Flycatcher	*Rhinomyias umbratilis*					/						NT
	White-rumped Shama	*Copsychus malabaricus*	/	/	/								LC
Monarchidae	Asian Paradise Flycatcher	*Terpsiphone paradisi*			/								LC
Ardeidae	Little Egret	*Egretta garzetta*								/	/	/	LC
	Intermediated Egret	*Ardae intermediare*					/			/	/		LC
	Great Egret	*Ardae alba*				/				/	/	/	LC
	Cattle Egret	*Bubulcus ibis*								/			LC
	Striated Heron	*Butorides striata*				/							LC
	Cinnamon Bittern	*Ixobrychus cinnamomeus*								/			LC
Accipitriidae	Black-shouldered Kite	*Elanus axillaris*				/							LC
	Crested Serpent-eagle	*Spilornis cheela*	/										LC
	Lesser Fish Eagle	*Icthyophaga humilis*		/	/								LC
	Crested Goshawk	*Accipiter trivirgatus*				/							LC
	Changeable Hawk Eagle	*Nisaetus cirrhatus*	/										LC
Columbidae	Spotted Dove	*Spilopelia chinensis*	/	/	/	/	/	/	/	/		/	LC
	Emerald Dove	*Chacophaps indica*		/									LC
	Pink-necked Green-pigeon	*Treron vernans*				/						/	LC
	Green Imperial Pigeon	*Ducula aenea*	/	/	/	/							LC
Cisticolidae	Ashy Tailorbird	*Orthotomus ruficeps*	/	/	/	/	/	/	/	/	/	/	LC
	Rufous-tailed Tailorbird	*Orthotomus sericeus*	/		/	/	/	/	/	/	/	/	LC
	Dark-necked Tailorbird	*Orthotomus atrogularis*	/	/	/	/							LC
	Yellow-bellied Prinia	*Prinia flaviventris*	/	/	/	/	/	/	/	/	/	/	LC
Cuculidae	Plaintive Cuckoo	*Cacomantis merulinus*	/	/	/		/						LC
	Greater Coucal	*Centropus sinensis*	/	/	/		/			/	/	/	LC
	Raffles’s Malkoha	*Rhinortha chlorophaea*	/	/	/								LC
	Chestnut-breasted Malkoha	*Phaenicophaeus curvirostris*		/	/								LC
Alcedinidae	Stork-billed Kingfisher	*Pelagopsis capensis*	/	/	/	/				/	/		LC
	Blue-eared kingfisher	*Alcedo meninting*	/			/				/	/		LC
	Oriental Dwarf-kingfisher	*Ceyx erithaca*	/	/									LC
Estrildidae	Dusky Munia	*Lonchura fuscans*				/				/	/	/	LC
	Chestnut Munia	*Lonchura atricapilla*								/	/	/	LC
Eurylaimidae	Banded Broadbill	*Eurylaimus harterti*				/							LC
	Black and Yellow Broadbill	*Eurylaimus ochromalus*		/									LC
Laniidae	Long-tailed Shrike	*Lainus schach*	/	/									LC
	Tiger Shrike	Lainus tigrinus						/	/				LC
Corvidae	Slender-billed Crow	*Corvus enca*	/	/	/	/	/						LC
Megalaimidae	Red-throated Barbet	*Psilopogon mystacophanos*			/								NT
Alcippeidae	Brown Fulvetta	*Alcippe brunneicauda*					/						NT
Vangidae	Black-winged Flycatcher-shrike	*Hemipus hirundinaceus*				/							LC
Campephagidae	Lesser Cuckooshrike	*Lalage fimbriata*		/									LC
Falconidae	Black-thighed Falconet	*Microhierax fringillarius*				/							LC
Anhingidae	Oriental Darter	*Anhinga melanogaster*				/							NT
Aegithinidae	Green Iora	*Aegithina viridissima*										/	NT
Rallidae	White-breasted Waterhen	*Amaurornis phoenicurus*	/	/		/	/	/		/	/	/	LC
Psittaculidae	Long-tailed Parakeet	*Belocercus longicaudus*	/	/	/	/							VU
Meropidae	Blue-throated Bee-eater	*Merops viridis*	/	/	/	/					/		LC
Coraciidae	Orienatl Dollarbird	*Eurystomus orientalis*	/	/		/							LC
Bucerotidae	Black Hornbill	*Anthracoceros malayanus*	/	/	/	/							VU
Trogonidae	Diard’s Trogon	*Harpactes diardii*			/								NT
Rhipiduridae	Malaysian Pied Fantail	*Rhipidura javanica*	/	/	/	/	/	/	/	/	/	/	LC
